# Value, Structure, and Curriculum in US Graduate Health Informatics Programs: Cross-Sectional Study

**DOI:** 10.2196/87479

**Published:** 2026-05-01

**Authors:** Suhila Sawesi, Diane M Dolezel, Pranitha Presingu, Michael Irungu

**Affiliations:** 1 Health and Bioinformatics Program, Department of Information Sciences and Technologies (IST), College of Computing, Grand Valley State University, 333 Michigan Street NE, Grand Rapids, MI, 49503, United States, 1 616-331-7827 ext 17827; 2 Department of Health Informatics & Information Management, Texas State University, Round Rock, TX, United States

**Keywords:** health informatics, education, curriculum, accreditation, Human Capital Theory, graduate, tuition

## Abstract

**Background:**

Graduate health informatics programs in the United States differ widely in cost, curriculum, and program design. However, it is unclear how these differences influence affordability, accreditation signaling, and preparation for a data-driven workforce.

**Objective:**

This study aimed to evaluate the value (tuition and affordability), structure (delivery format, credit load, culminating experience, and accreditation), and curriculum (technology content emphasis) of US graduate health informatics programs. It examined how accreditation and modality relate to program design, and whether tuition-normalized curriculum breadth differed by accreditation status.

**Methods:**

A cross-sectional study of 107 US graduate health informatics programs was conducted using publicly available data collected between January and May 2025. Tuition was standardized to cost per credit. Curricular content was coded for technology density and mapped to the Commission on Accreditation for Health Informatics and Information Management Education domains. Comparative statistics, regression models, and exploratory cluster analyses were used to assess relationships between tuition, credit requirements, accreditation, delivery format, and curriculum characteristics.

**Results:**

Programs varied by delivery format, with 37 of 107 (34.6%) online, 32 of 107 (29.9%) hybrid, 23 of 107 (21.5%) in person, and 15 of 107 (14.0%) flexible. Credit requirements most commonly fell between 31 and 39 credits. Culminating experiences included capstone (54/107, 50.5%), internships (21/107, 19.6%), and thesis (7/107, 6.5%). Required credit hours showed modest variation by delivery format but not by accreditation status. Accreditation was not associated with differences in the tuition-normalized curriculum breadth structural proxy in this program-level analysis. Programs requiring internships had significantly higher mean credit loads than programs without internships (39.0 vs 31.3 credits; *P*=.005). Cluster analysis revealed 4 descriptive program configurations differentiated by cost, modality, credit requirements, and culminating experiences.

**Conclusions:**

In this program-level descriptive analysis, accreditation status was not associated with differences in tuition-normalized curriculum breadth structural proxy. Instead, delivery format and internship requirements were descriptively associated with variation in credit load and cost. Improving transparency in tuition models and aligning program structure with curricular scope may support efforts to enhance equity and value in graduate health informatics education.

## Introduction

### Background and Conceptual Framework

Graduate education in health informatics is evolving amid continued discussion regarding affordability, quality assurance, and measurable returns on investment. While undergraduate tuition growth has slowed, graduate education remains costly, particularly in health informatics, where financial liabilities can be substantial and returns unpredictable. Recent federal regulations mandating program-level disclosure of student debt and earnings have intensified attention to tuition structures, delivery modalities, and return on investment [1-3].

**Figure 1.**
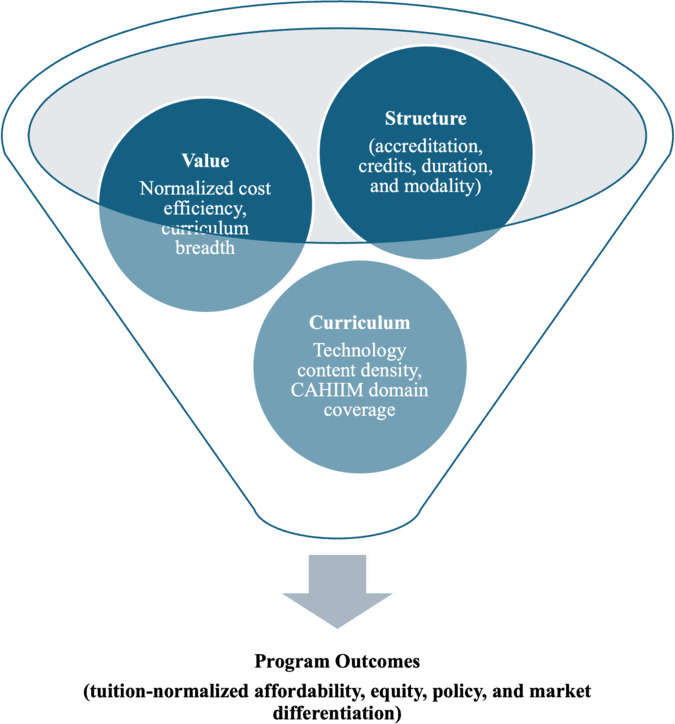
Conceptual framework: value, structure, and curriculum. This framework, grounded in Human Capital Theory, categorizes educational investment inputs into 3 core domains: Value (tuition-normalized curriculum breadth and credit-based costs), Structure (accreditation status, credit requirements, delivery modality, and program duration), and Curriculum (technology content density and CAHIIM domain coverage). These structural domains are analyzed as correlates of program-level outcomes, including affordability and market differentiation. CAHIIM: Commission on Accreditation for Health Informatics and Information Management Education.

This study is grounded in Human Capital Theory (HCT) [4], which conceptualizes education as an investment that builds skills and yields economic returns. Within this framework (Figure 1), tuition represents the investment cost of acquiring human capital (Value), accreditation functions as a quality signal to the labor market (Structure), and curriculum reflects the skills and competencies that enhance employability (Curriculum). Together, these domains shape outcomes related to affordability, transparency, equity, market positioning, and labor-market returns (eg, debt-to-earnings ratios). While this study uses HCT to categorize educational inputs, we do not measure actual labor market outcomes. In this context, HCT serves as a conceptual lens to evaluate the structural components of educational investment rather than as a framework for measuring postgraduation economic returns.

### Affordability and Delivery Modalities

Affordability discussions have shifted from headline tuition to a broader notion of value. Although undergraduate prices have decelerated, graduate programs continue to present affordability challenges due to high net prices and debt loads [1]. The 2023 Financial Value Transparency and Gainful Employment regulations require institutions to disclose program-level debt and earnings, sharpening scrutiny of tuition models and return on investment [2].

Modality further complicates costs: despite advertised uniform tuition across online, in-person, and hybrid formats, hidden or modality-specific fees can raise total expenses, with some evidence of an “online premium” that elevates all-in costs for distance learners [5]. At the same time, the credit hour, linking cost to program duration, remains contested. Federal definitions tie it to student effort and outcomes, whereas critics argue that reliance on the Carnegie Unit constrains competency-based and flexible models [5,6]. Despite heightened policy focus, comparative research remains scarce on how tuition per credit, credit loads, and delivery modality interact with accreditation across graduate health informatics programs.

### Quality Assurance and Curricular Content

Quality assurance adds a second dimension. The Commission on Accreditation for Health Informatics and Information Management Education (CAHIIM) sets accreditation standards, while the American Medical Informatics Association (AMIA) defines master’s-level core competencies [7,8]. Internationally, the International Medical Informatics Association has issued education recommendations (2010; updated 2023) that map skill domains to program design across degree levels [9,10]. However, it is unclear whether accreditation aligns with specific program structures (eg, credit loads, accelerated pathways, and culminating requirements) or with the technical content density needed for an artificial intelligence (AI)–driven and data-driven workforce.

Curricular content also varies widely across programs, further complicating the relationship between accreditation and workforce readiness. Prior work documents gaps in areas such as database management and analytics relative to employer expectations and AMIA competencies, as well as substantial heterogeneity across US biomedical and health informatics curricula [11-13]. Comparative analyses from other regions likewise show that certification alone does not guarantee consistent competency coverage, and multiple authors advocate for more applied, skills-based training aligned with workforce demands [13,14]. A recent scientometric review (2014‐2023) further highlights the field’s multidisciplinary evolution and shifting thematic emphases [15]. Yet, little is known about how curriculum design (eg, technology content density, CAHIIM, and domain coverage) interacts with pricing, modality, and accreditation, or whether programs can be grouped into configurations that integrate value and structural features.

### Study Goal and Objectives

To address these gaps, an HCT-informed framework comprising Value, Structure, and Curriculum (Figure 1) is applied to evaluate US graduate health informatics programs. Using publicly available program data, the study examines how interactions among tuition, modality, and accreditation shape affordability; assesses whether curriculum design varies with technical content and accreditation; and identifies program configurations that integrate structural and value-based attributes. Findings are intended to describe the program-level investment landscape rather than to imply direct causal links to employment outcomes.

## Methods

### Study Design and Reporting

This cross-sectional, descriptive, comparative observational study evaluated US graduate (master’s level or equivalent) health informatics programs. Reporting followed the STROBE (Strengthening the Reporting of Observational Studies in Epidemiology) guideline for cross-sectional studies [16]. Eight prespecified research questions (RQ1-RQ8) examined credit-hour requirements, tuition-normalized curriculum breadth, accelerated pathways, tuition stratification, technology content density, internship requirements, format-duration patterns, and descriptive program configurations.

### Eligibility and Program Selection

The unit of analysis was the graduate program. Eligible programs were US-based master’s or graduate-equivalent offerings in health, biomedical, or clinical informatics and related analytics programs. To ensure a transparent and replicable sample based solely on publicly available documentation, analytics-focused programs were deemed eligible if they met at least 1 of the following verifiable criteria:

1. *Institutional affiliation*: The degree was housed within a health-related college (eg, medicine, nursing, and public health) or an interdisciplinary technology-focused college (eg, computing, information sciences, or engineering).
2. *Explicit health designation*: The program title or official catalog description explicitly designated a “Health Informatics,” “Health Analytics,” or “Clinical Informatics” track, concentration, or specialization.
3. *Core curricular focus*: The publicly available course catalog listed core requirements or elective clusters dedicated to health sector applications (eg, “Healthcare Data Science” or “Clinical Decision Support”).

Programs were excluded if they were duplicates, suspended, or closed during the study window; located outside the United States; or limited to undergraduate or certificate credentials.

### Search Strategy and Data Sources

Programs were identified via 3 primary channels: CAHIIM directory, the AMIA institutional membership directory, and structured web searches finalized on May 15, 2025. To facilitate replication, web searches used the Google search engine with the following exact search strings:

1. “graduate health informatics programs US”
2. “health analytics masters US”
3. “biomedical informatics master's degrees United States”
4. “clinical informatics graduate curriculum US”

**Figure 2.**
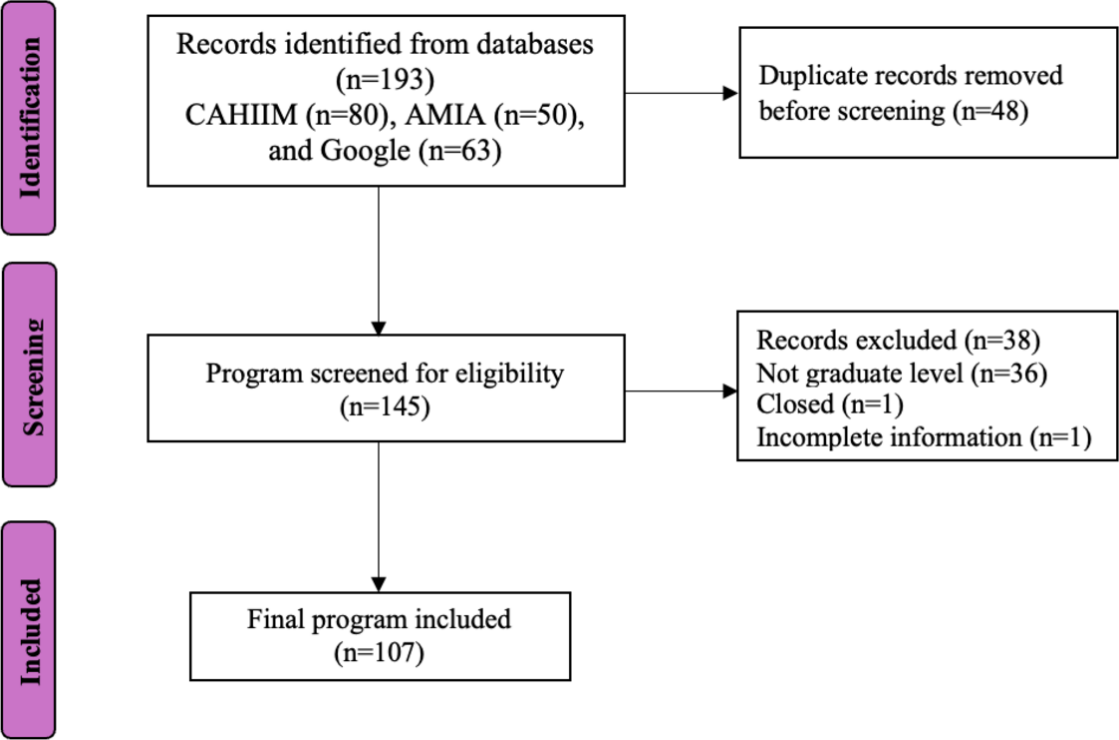
Flow diagram of the program identification and selection process. A total of 193 records were identified across CAHIIM (n=80), AMIA (n=50), and Google searches (n=63). Following the removal of duplicates (n=45), 145 programs were screened. Thirty-eight programs were excluded (36 not graduate level, 1 closed, and 1 with incomplete data), resulting in a final analytical sample of 107 programs. AMIA: American Medical Informatics Association; CAHIIM: Commission on Accreditation for Health Informatics and Information Management Education.

Search results were restricted to the first 100 hits per string. For each program, the most recent institutional catalog (2024‐2025 or 2025‐2026) and program-specific web pages were used as the primary data sources. In cases of conflicting information, the official university catalog was prioritized. The identification, screening, and inclusion process are summarized in the study flow diagram (Figure 2).

### Variables Coding for Value and Structure

#### Overview

A total of 107 programs across 60 institutions were coded across 18 fields spanning institutional identity, program descriptors (title, delivery format, and nominal duration), academic requirements (total credits, tracks, and course classification), culminating experiences (capstone, thesis, and internship; required or optional), structural attributes (accelerated pathways and professional science master’s designation), financial or support (tuition per credit [US dollars], F-1 visa eligibility), and accreditation (CAHIIM status or notes). Abbreviations are provided in Multimedia Appendix 1; definitions and the variable framework are detailed in Multimedia Appendix 2; and data sources are listed in Multimedia Appendix 3. Programs labeled “Flexible,” indicating multiple delivery options, were coded accordingly.

#### Accreditation Coding

CAHIIM accreditation was coded at the *program and degree levels,* rather than at the institutional or departmental level. A program was classified as CAHIIM-accredited only if the specific graduate degree under analysis (eg, MS in Health Informatics or equivalent) was explicitly listed as actively accredited in the CAHIIM program directory during the study window (January-May 2025). Programs housed within departments offering other CAHIIM-accredited degrees (eg, Health Information Management) were not classified as accredited unless accreditation explicitly applied to the analyzed program. Programs in candidacy, transition, or legacy status were also excluded from the accredited category.

Technology content density was calculated as a simple count of predefined technology-related keywords found in course titles and descriptions for required and elective courses. All keywords were weighted equally and counted only once per program to prevent duplication from repeated course descriptions. Keywords were selected a priori based on commonly cited technical skill areas in graduate health informatics education (eg, analytics, databases, AI, and cybersecurity) and were subsequently mapped to CAHIIM domains for standardized classification. While catalog descriptions do not provide the granular detail of a full syllabus, they serve as a reliable proxy for identifying a program’s primary curricular pillars and intended technical emphasis. Mapping was restricted to explicit mentions of technology areas to ensure high-level domain alignment without overinferring specific lesson content. Total keyword counts per program ranged from 0 to 15 and were used to compare overall levels of technical focus. We acknowledge that keyword-based density serves as a measure of curricular emphasis and intent; it is not a validated measure of actual skill acquisition or syllabus-level depth.

To better reflect differential curricular emphasis between required and elective coursework, we constructed a weighted technology content density sensitivity metric. Keywords identified in required (core) courses were assigned a weight of 2, whereas keywords appearing only in elective courses were assigned a weight of 1. Keywords were additionally grouped a priori into structured technical domains (eg, AI or machine learning, data engineering or database systems, analytics or statistics, cybersecurity or information assurance, and clinical informatics systems) to align the metric with standardized curricular categories. The weighted density score for each program was calculated as the sum of weighted domain occurrences, counted once per domain per program to avoid duplication. Primary analyses used the unweighted density metric; weighted results were examined as sensitivity analyses to assess robustness.

### Operationalization of the Value-Structure-Curriculum Framework

The framework was operationalized to evaluate the structural components of the educational investment:

1. *Value*: Defined by tuition per credit and tuition-normalized curriculum breadth (US dollars per credit per distinct CAHIIM-aligned domain). As a structural proxy, this metric assumes equal weighting across domains and is intended to compare pricing intensity relative to curricular scope rather than measuring instructional depth or student outcomes.
2. *Structure*: Comprised delivery format (online, in person, hybrid, and flexible), required credits, nominal duration, culminating experiences, accelerated pathways (eg, 3+2 and 4+1), and CAHIIM accreditation.
3. *Curriculum*: Assessed via technology-content density (eg, AI, machine learning, data science, and cybersecurity) and CAHIIM domain coverage (binary flags).

### Derived Measures

Total program cost was calculated as tuition per credit × required credits. The tuition-normalized curriculum breadth metric was defined as (US dollars per credit) divided by the number of domains (eg, US $800 per credit across 10 domains → US $80 per credit per domain). The metric was intentionally specified independent of required credit hours, which were analyzed separately. Sensitivity analyses contrasted required-only vs required + elective domain coverage.

### Data Cleaning and Preprocessing

Standardization steps included categorical harmonization (eg, No/None/blank → No), text normalization, tuition normalization to per credit (including mandatory program or online fees explicitly tied to credits; excluding optional fees), and reconciliation of credit ranges using midpoints. All transformations were scripted and reproducible. The code list is provided in Multimedia Appendix 2.

### Interrater Reliability

Two researchers (PP and MI) independently piloted 20 programs. Agreement for key fields (format, credits, internship requirement, accreditation, and tuition tier) was assessed using Cohen κ (or Gwet’s AC1 for sparse categories); discrepancies were adjudicated and coding rules updated. The remaining 87 programs were single-coded with adjudication as needed. Final κ/AC1 values are reported in Multimedia Appendix 3.

### Bias, Missing Data, and Sensitivity

Information bias was mitigated through multisource verification, web archiving, and conservative tuition rules. The risk of selection bias due to limited online presence was reduced using CAHIIM and AMIA seeding and structured web searches. Missing data were coded as NA (not available). Primary analyses used complete cases with denominators reported. Sensitivity checks repeated analyses after (1) excluding programs with unclear or mixed tuition and (2) restricting the sample to programs with dated catalogs.

### Statistical Analysis

Analyses were conducted in R (version 4.4.1; R Core Team) (Multimedia Appendix 4 lists packages). Given the descriptive, observational nature of the dataset, statistical tests were used to identify potential associations and characterize patterns rather than infer causal relationships. All tests were 2-sided with α=.05. To assess the practical significance, we report effect sizes, including partial eta-squared *(*η_p_²) for ANCOVA and ANOVA models and Cohen *d* or *r* for group comparisons, along with 95% CIs for mean differences. For related test families, Tukey or Games-Howell procedures were used to control the family-wise error rate; otherwise, Benjamini-Hochberg–adjusted *q* values are reported.

Descriptives include counts and percentages for categorical variables, as well as medians (IQR) and means (SD) for monetary values. Group comparisons for tuition and credit hours used Welch ANOVA (with nonparametric alternatives, Mann-Whitney *U* [Wilcoxon rank sum] tests, when distributions were nonnormal). Categorical comparisons used chi-square or Fisher exact tests, as appropriate. Credit-hour models used ANCOVA or ANOVA with format and accreditation as factors and tuition per credit as a covariate; assumptions were checked, with robust methods and bootstrap 95% CIs (2000 resamples) when violated. Tuition-normalized curriculum breadth was compared with Wilcoxon rank sum tests and visualized with median or density plots. Technology alignment was assessed via linear regression modeling content density as a function of tuition per credit and total credits, with accreditation and interaction terms; we report standardized coefficients (*β*), SEs, 95% CIs, *P* values, and *R*². Results near the *P*=.05 threshold are interpreted as descriptive patterns requiring further validation.

Clustering was conducted as an exploratory clustering analysis to uncover descriptive program configurations. Continuous variables were *z*-score standardized, categorical variables were one-hot encoded, and culminating experiences were ordinally coded. *k*-means was used as the primary method for its interpretability, with Ward hierarchical clustering applied as a robustness check to ensure partition stability. The number of clusters was selected using elbow, silhouette, and Calinski-Harabasz indices, with bootstrap resampling used to assess stability. We emphasize that clusters with very small membership (n

≤4) are rare configurations and are presented solely for descriptive completeness rather than generalizable inference.

External validity was examined through differences in accreditation status and tuition- and credit-based metrics. Results are interpreted as descriptive configurations rather than definitive segments. Although Euclidean *k*-means is not optimal for nominal data, alternative mixed-type methods (eg, Gower distance with *k*-medoids or *k*-prototypes) were considered; *k*-means was selected to prioritize comparability and interpretability in this education policy context, with multimethod checks used to mitigate bias.

### Ethical Considerations

All data analyzed in this study were obtained from publicly available institutional and program websites. In limited cases where online information was ambiguous, program offices were contacted solely to confirm publicly applicable program details (eg, tuition rates or required credit hours). No confidential, unpublished, proprietary, or student-level information was requested, accessed, or used. This study was reviewed by the Grand Valley State University Institutional Review Board (IRB) and determined to be not human subjects research and therefore not requiring IRB approval, in accordance with federal regulations (45 CFR 46.102) (IRB determination letter, April 2, 2026).

## Results

### Descriptive Overview of Programs

A total of 107 graduate health informatics programs from 60 US institutions were identified and analyzed. Multimedia Appendix 5 summarizes their structural, curricular, and financial characteristics. Health Informatics (33/107, 30.8%) was the most common program designation, followed by Biomedical Informatics (18/107, 16.8%). Program delivery formats differed, with online programs most common (37/107, 34.6%), followed by hybrid (32/107, 29.9%), in person (23/107, 21.5%), and flexible formats (15/107, 14.0%), where flexible denotes programs that provide more than one delivery option.

The typical program duration was 19‐24 months (72/107, 67.3%), with credit requirements most frequently falling between 31 and 39 hours (53/107, 49.5%). Roughly one-third of programs (35/107, 32.7%) required prerequisites. Culminating experiences included capstone projects (54/107, 50.5%), internships (21/107, 19.6%), and theses (7/107, 6.5%). Tuition per credit showed substantial variation: 39.3% of programs charged US $500 to US $999, while 13.1% exceeded US $2000. CAHIIM accreditation, when defined at the program-specific degree level, was present in 33 of 107 programs (30.8%).

### Policy and Cost Dynamics

#### RQ1: Tuition Costs, Program Format, and Accreditation

We tested whether tuition per credit hour, program format, and CAHIIM accreditation status were associated with graduate program credit hour requirements. Descriptive statistics stratified by format and accreditation are shown in Tables1  2.

**Table 1.** Tuition and credit hours by program format and accreditation status (N=107)^a^.

Program format	CAHIIM^b^ accredited	Values, n (%)	Mean tuition, US $	SD tuition, US $	Mean credits	SD credits
Online	Yes	20 (18.7)	699.16	286.62	33.43	8.76
	No	17 (15.9)	368.90	323.68	24.15	9.89
In person	Yes	2 (1.9)	270.76	306.47	33.50	11.91
	No	21 (19.6)	536.14	785.55	34.67	12.65
Hybrid	Yes	7 (6.5)	392.53	313.39	36.36	5.44
	No	25 (23.4)	470.13	469.03	33.89	7.82
Flexible^c^	Yes	4 (3.7)	375.00	335.21	33.68	11.96
	No	11 (10.3)	404.37	389.13	34.20	12.48

a Values rounded to 2 decimals. Percentages calculated from total N=107.

b CAHIIM: Commission on Accreditation for Health Informatics and Information Management Education.

c Flexible denotes programs providing more than 1 delivery option.

**Table 2.** Effect size and practical significance of program format on credit requirements.

Comparison	Adjusted mean difference	95% CI	Effect size (η_p_²^a^ or *d*^b^)
Main effect: format	—^c^	—	η_p_²=0.076 (moderate)
Maximum vs minimum comparison^d^	9.7 credits	2.1 to 17.3	*d*=0.82 (large)
Accreditation effect	0.15 credits	−3.2 to 3.5	η_p_²<0.001 (negligible)

a Partial eta-squared.

b Cohen *d*.

c Not available.

d Comparison between Hybrid and Online nonaccredited programs.

Online accredited programs comprised the largest subgroup (20/107, 18.7%), with a mean tuition of US $699.16 (SD US $286.62) per credit and an average of 33.43 (SD 8.76) credit hours. Online nonaccredited programs (17/107, 15.9%) had lower mean tuition (US $368.90, SD US $323.68) and the fewest required credit hours (mean 24.15, SD 9.89 hours). In person accredited programs were rare (2/107, 1.9%), with a mean tuition of US $270.76 (SD US $306.47) and an average of 33.5 credits (SD 11.91).

A two-way ANCOVA with tuition as a covariate suggested that program format was associated with required credit hours (*F*_3,98_=2.70; *P*=.049; *η_p_*²*=.*076). The adjusted mean difference between the format with the highest requirement (Hybrid) and the lowest (Online nonaccredited) was 9.7 credit hours (95% CI 2.1‐17.3), representing a moderate practical difference of approximately 3 standard courses. Consistent with our exploratory approach, this result is interpreted as a descriptive pattern rather than a definitive predictor.

By contrast, accreditation status showed a negligible effect on credit requirements (*P*=.94; *η_p_²*<.001), with a mean difference of only 0.15 credits (95% CI −3.2 to 3.5). Specifically, neither tuition per credit (*P*=.17), accreditation status (*P*=.94), nor the format× accreditation interaction (*P*=.43) reached statistical significance (Multimedia Appendix 6).

**Figure 3.**
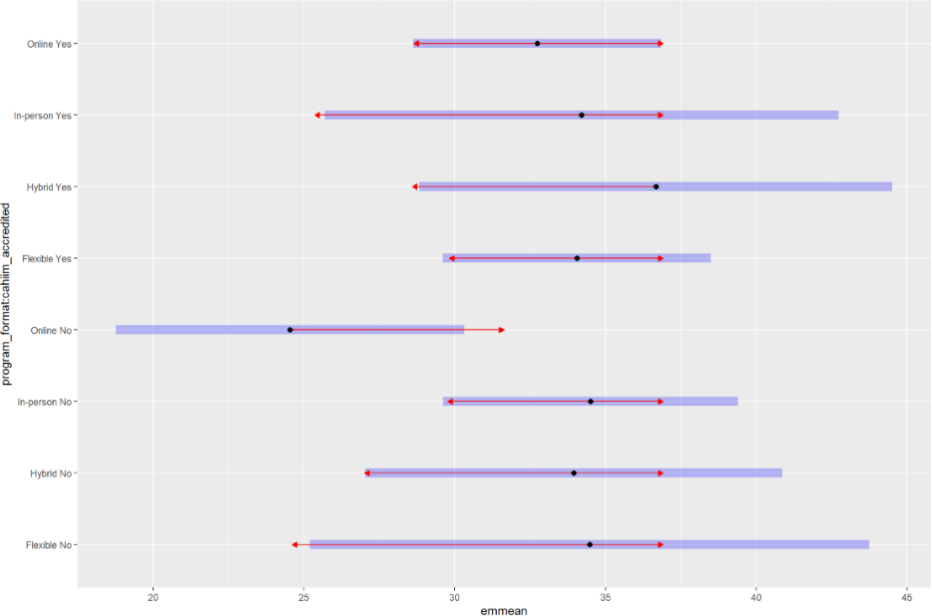
Estimated marginal means of credit hours by program format and accreditation status. Visualization of adjusted credit-hour requirements based on a two-way ANCOVA. Estimated marginal means and 95% CIs are provided for graduate health informatics programs stratified by delivery format and Commission on Accreditation for Health Informatics and Information Management Education accreditation. Descriptive patterns indicate that online nonaccredited programs generally require fewer credits, whereas other modalities cluster between 33 and 36 credit hours. Points represent estimated marginal means, blue horizontal bars indicate 95% CIs, and red arrows denote pairwise comparisons between group means.

Post hoc Tukey pairwise comparisons in MultimediaAppendices 7  8 suggested that online nonaccredited programs had lower credit requirements than in person nonaccredited (Δ=10.52; *P*=.17) and hybrid nonaccredited programs (Δ=9.74; *P*=.44), but none reached significance after adjustment. Estimated marginal means and 95% CIs are shown in Figure 3, which highlights clustering of in person, hybrid, and flexible programs around 33‐36 credits, while online nonaccredited programs averaged fewer credits.

#### RQ2: Tuition-Normalized Curriculum Breadth (US Dollars per Credit per CAHIIM Domain)

We calculated a tuition-normalized curriculum breadth metric as tuition per credit divided by the number of distinct CAHIIM-aligned domains.

Because distributions were nonnormal for both accredited and nonaccredited groups (Shapiro-Wilk: accredited *W*=0.72, *P*<.001; nonaccredited *W*=0.60, *P*<.001), group comparisons were performed using the Wilcoxon rank sum test.

**Figure 4.**
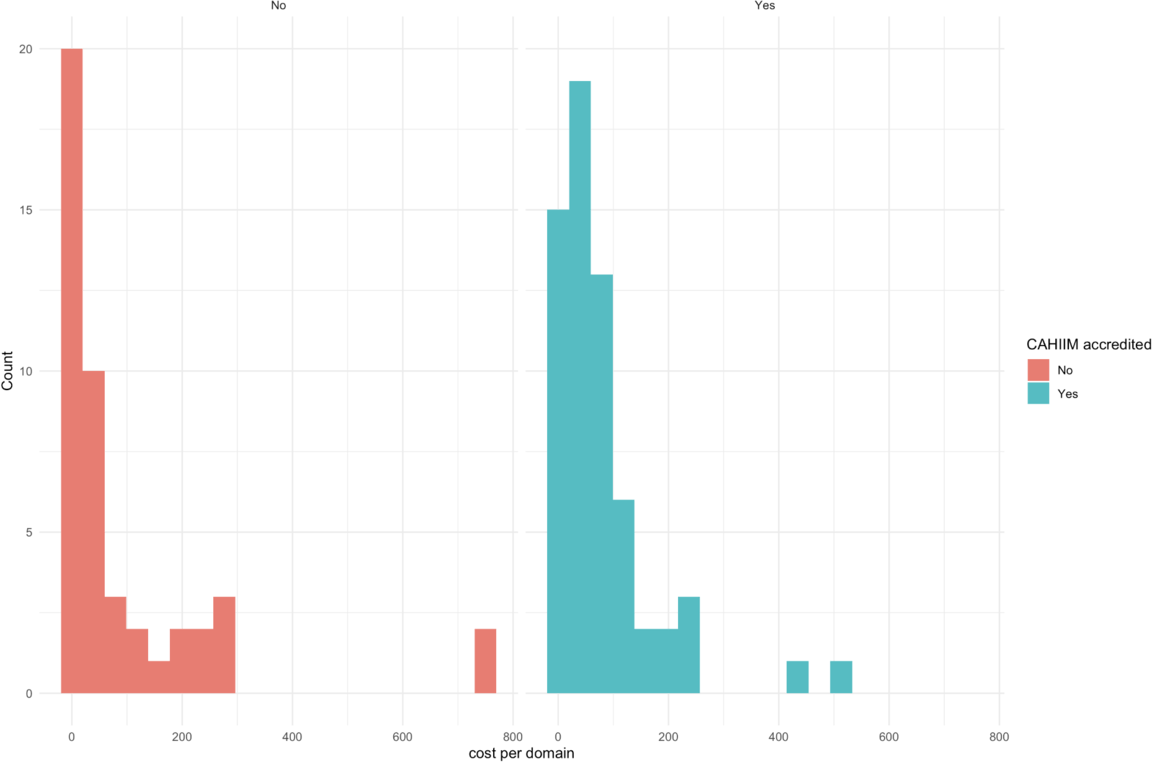
Distribution of tuition‑normalized curriculum breadth (US dollars per credit per domain) by accreditation status. Histograms show the distribution of tuition‑normalized curriculum breadth metric across accredited (blue) and nonaccredited (red) programs. The metric represents the ratio of tuition per credit to the number of CAHIIM-aligned domains. Accredited programs demonstrate tighter clustering, while nonaccredited programs show higher variability; however, group medians were not statistically different (*P*=.35). CAHIIM: Commission on Accreditation for Health Informatics and Information Management Education.

Across 107 programs with complete data, CAHIIM-accredited programs (n=33) had a mean tuition-normalized curriculum breadth score of 77.22 (median 48.80, SD 96.48), compared with 94.71 (median 40.50, SD 166.57) among nonaccredited programs (n=74). No statistically significant difference was observed between accredited and nonaccredited programs for this tuition-normalized curriculum breadth metric (*W*=1247, *P*=.35) (Table 3). As shown in Figure 4, accredited programs clustered more tightly around the median, whereas nonaccredited programs exhibited greater variability, including several outliers, but with comparable central tendencies. This indicates that, within this sample, accreditation status was not associated with differences in this structural pricing-to-breadth proxy.

**Table 3.** Tuition-normalized curriculum breadth (US dollars per credit per Commission on Accreditation for Health Informatics and Information Management Education domain) by accreditation status^a^.

Accreditation status	Values, n	Mean	Median	SD
Yes	33	77.22	48.8	96.48
No	74	94.71	40.5	166.57

a Wilcoxon rank sum test: *W*=1247; *P*=.35. Normality: Shapiro-Wilk (accredited: *W*=0.72, *P*<.001; nonaccredited: *W*=0.60, *P*<.001).

#### RQ3: Tuition Costs and Accelerated Pathways by Accreditation

We next examined whether accelerated pathways were associated with lower tuition per credit and whether this relationship differed by accreditation status. As shown in Multimedia Appendix 9, accelerated programs tended to exhibit lower tuition distributions compared with nonaccelerated programs. Stratification by CAHIIM accreditation revealed a more nuanced interaction (Multimedia Appendix 10): while accredited programs overall had higher tuition values, accredited accelerated programs showed reduced tuition compared with their nonaccelerated counterparts.

Levene’s test indicated significant heterogeneity of variance across the 4 groups (*F*_3,103_=5.47, *P*=.002). A two-way ANOVA confirmed significant main effects of acceleration (*F*_1,103_=17.88, *P*=.001) and accreditation (*F*_1,103_=10.92, *P*=.002), as well as their interaction (*F*_1,103_=7.25, *P*=.005). These findings suggest that the cost benefits of acceleration are moderated by accreditation status.

### Curriculum and Cost Alignment

#### RQ4: Tuition Stratification by Credit Load, Format, and Accreditation

We next examined whether tuition per credit varied by program format (Flexible, Hybrid, In-person, and Online) and CAHIIM accreditation status. A two-way ANOVA including all programs with complete data (n=103) did not detect statistically significant effects: program format (*F*_3,99_=1.33, *P*=.27), accreditation (*F*_1,99_=0.39, *P*=.54), and the format × accreditation interaction (*F*_3,99_=2.05, *P*=.111). Tukey-adjusted pairwise comparisons were also nonsignificant (MultimediaAppendices 7  11).

Descriptive statistics are reported in Table 4. Among accredited programs, mean tuition per credit was highest for online programs (20/107, 18.7%; mean US $699.16, SD US $286.62) and lowest for in-person programs (2/107, 1.9%; mean US $270.76, SD US $306.47). Nonaccredited programs showed the opposite pattern, with in-person programs (21/107, 19.6%) averaging the highest tuition (mean US $536.14, SD US $785.55) and online programs (17/107, 15.9%) the lowest (mean US $368.90, SD US $323.68).

**Table 4.** Tuition per credit by program format and accreditation (N=107)^a^.

Program format	Accreditation	Mean tuition (SD), US $		Values, n (%)
Flexible	Yes	374.99 (335.21)		4 (3.4)
	No	404.37 (389.13)		11 (10.3)
Hybrid	Yes	392.53 (313.39)		7 (6.5)
	No	470.13 (469.03)		25 (23.4)
In-person	Yes	270.76 (306.47)		2 (1.9)
	No	536.14 (785.55)		21 (19.6)
Online	Yes	699.16 (286.62)		20 (18.7)
	No	368.90 (323.68)		17 (15.9)

a Values for n (%) derived from the total sample distribution.

The interaction pattern is illustrated in Multimedia Appendix 12, which plots mean tuition per credit by program format and accreditation. Accredited online programs stand out as costlier, whereas nonaccredited in-person programs are relatively more expensive.

#### RQ5: Tuition Costs and Credit Loads in Relation to Technology Content Density and Accreditation

Pearson correlation tests indicated that tuition per credit was not significantly correlated with technology content density (*r*=−0.08, *P*=.39; 95% CI −0.27 to 0.11). By contrast, technology content density—representing the explicit mention of technical keywords in course descriptions—showed a small positive correlation with average credit requirements (*r*=0.21, *P*=.03; 95% CI 0.03-0.39).

To examine whether accreditation moderated these relationships, regression models were estimated with interaction terms. In the tuition model (*R*²=0.03, *P*=.39), neither technology density (*β*=–10.25, *P*=.56) nor accreditation (*β*=–120.59, *P*=.43) significantly predicted tuition, and the interaction effect was nonsignificant (*β*=−1.24, *P*=.96).

In the credit load model (*R*²=.09, *P*=.03), technology density positively predicted higher credit requirements (*β*=1.16, *P*=.005). Accreditation status was not significantly associated with higher credit averages (*β*=7.77, *P*=.39). The interaction of technology density × accreditation was not statistically significant (*β*=−.88, *P*=.11), suggesting that the relationship between technology density and credit load was similar across accredited and nonaccredited programs. We note that these findings reflect curricular emphasis in public documentation rather than a validated measure of student skill acquisition. Full regression outputs appear in Multimedia Appendix 13. Sensitivity analyses using the weighted technology density metric prioritizing required over elective coursework produced similar associations with credit requirements and no relationship with tuition per credit, indicating robustness of the primary findings.

#### RQ6: Internship Requirement and Program Costs

To assess whether mandatory internships are associated with differences in tuition or credit requirements, we compared programs with and without an internship requirement. Among the 107 programs analyzed, 21 of 107 (19.6%) required internships, while 86 of 107 (80.4%) did not.

A Shapiro-Wilk test confirmed nonnormality of tuition data (*W*=0.81, *P*<.001), so Mann-Whitney *U* tests were applied. There was no significant difference in tuition between internship-required and noninternship programs (*W*=796, *P*=.40). However, programs with mandatory internships required significantly more credit hours (mean 39.0, SD 8.1) than those without (mean 31.3, SD 10.7; *W*=549; *P*=.005), with a small to moderate effect size (*r*=0.27). These differences are visualized in Multimedia Appendix 14, which shows a tighter clustering of higher credit requirements among internship-required programs compared with noninternship programs.

### Program Structures and Configurations

#### RQ7: Structure Patterns—Program Format and Duration

**Figure 5.**
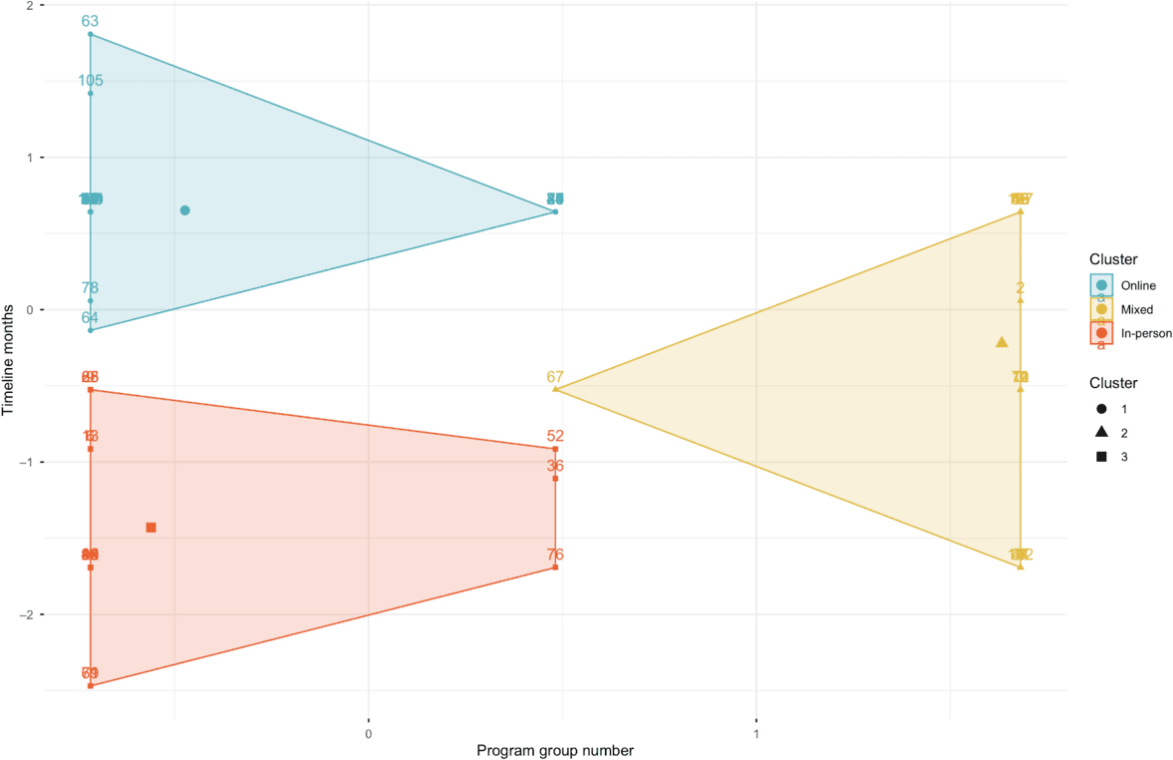
*K*-means clustering of program format and timeline (3-cluster solution). Scatterplot identifying 3 descriptive delivery duration configurations: Cluster 1 (blue, Online) represents shorter programs with modest variance, cluster 2 (yellow, Mixed: Hybrid/Flexible) includes standardized programs centered on a 24-month duration, and cluster 3 (red, In-person) reflects intermediate-length programs with high variability. Shaded regions represent cluster boundaries; note that small subclusters (n≤4) are rare configurations within this sample.

Across formats (Flexible, Hybrid, In-person, and Online), the most common duration was 24 months (Multimedia Appendix 8). A format × duration crosstab (months coded at 8, 12, 15‐16, 18, 20‐21, 24, and 28‐30) indicated no significant association between format and duration (*χ*²_27_=24.76, *P*=.59). Full results appear in Multimedia Appendix 15; patterns are illustrated in MultimediaAppendices 5  16. To examine continuous variation, we conducted a *k*-means cluster analysis on standardized program format and timeline (months). The elbow method supported a 3-cluster solution, which produced 3 well-separated clusters (Figure 5).

1. Cluster 1 Online (37/107, 34.6%): this cluster represents all online programs (20 accredited and 17 nonaccredited), typically characterized by shorter durations (mean 13.3, SD 2.95 months).
2. Cluster 2 Mixed (Hybrid/Flexible) (47/107, 43.9%): comprising 32 hybrid and 15 flexible programs, this group featured the most standardized timelines centered on approximately 24 months (mean 24.1, SD 1.15 months).
3. Cluster 3 In-person (23/107, 21.5%): consisting of 2 accredited and 21 nonaccredited programs, these programs showed intermediate length but higher variability (mean 19.6, SD 4.98 months).

Taken together, the chi-square analysis suggests no categorical dependency, whereas the clustering reveals 3 coherent delivery-duration configurations: (1) shorter Online programs, (2) standardized 2-year Mixed programs, and (3) more variable In-person programs. Clusters with very small membership (n≤4), such as the accredited in-person subgroup, are reported as rare configurations and were not used for inferential group comparisons; their role remains descriptive and hypothesis-generating.

#### RQ8: Distinct Program Configurations and Accreditation Differences

**Figure 6.**
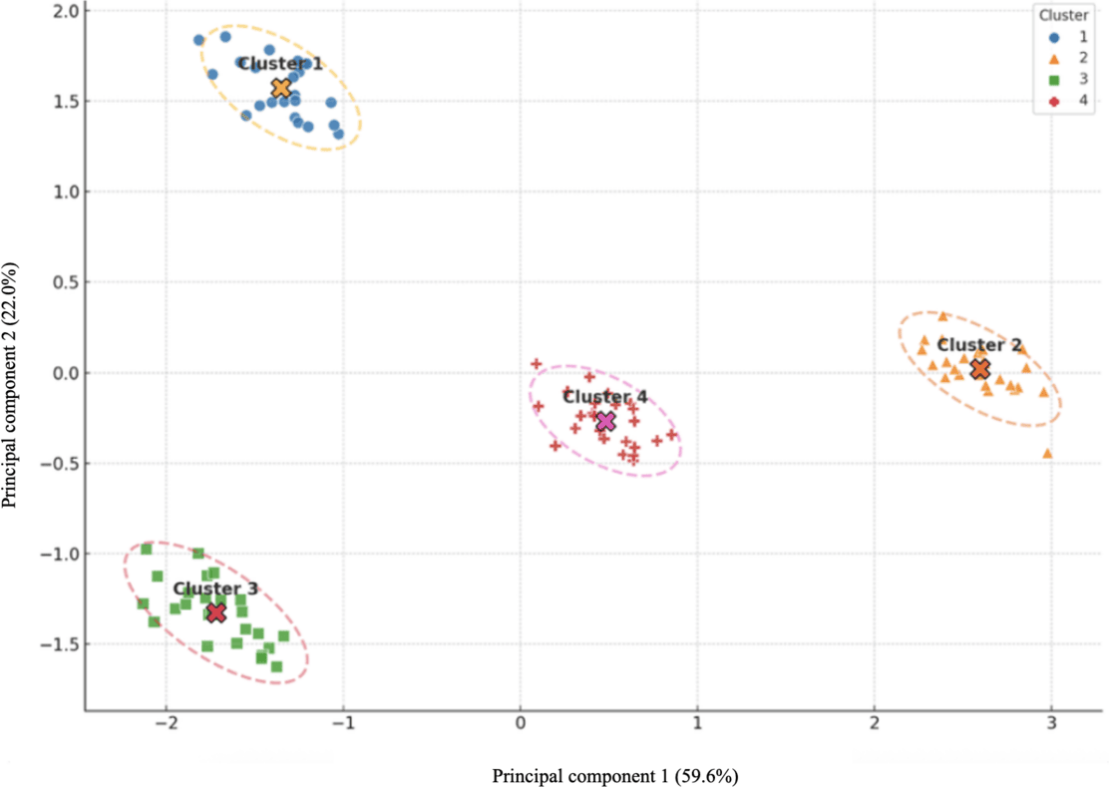
Principal component analysis (PCA) visualization of the *k*-means cluster solution (N=107). Four descriptive program configurations derived from *k*-means analysis, visualized in a 2D PCA space (PC1 explaining 59.6% and PC2 explaining 22.0% of variance). Ellipses denote 95% confidence regions for cluster 2 (flexible and low-cost) and cluster 4 (online and moderate-cost). Clusters 1 and 3 represent rare program configurations (n=4 and n=3, respectively) and are presented for descriptive completeness; centroids are marked by crosses.

Four clusters were identified using *k*-means clustering based on tuition per credit, credit requirements, program format, culminating experience, and track offerings. Clustering results are presented as descriptive patterning rather than definitive classification. The resulting clusters are visualized in Figure 6 and summarized in MultimediaAppendices 17  18. Points represent individual programs; ellipses show 95% confidence regions, with cluster centroids marked by crosses.

While 4 clusters were derived, cluster 1 (4/107, 3.7%) and cluster 3 (3/107, 2.8%) represent rare configurations within the sample and are presented for descriptive completeness rather than as generalizable program configurations.

1. Cluster 1 (4/107, 3.7%): This group consisted of hybrid programs with the highest average credit requirements (mean 43.2) and the highest tuition per credit (mean US $668). These programs most frequently offer both capstone and thesis options as culminating experiences. CAHIIM accreditation was present in 1/4 (25%) of programs.
2. Cluster 2 (46/107, 43.0%) primarily included flexible-format programs with the lowest average tuition (mean US $424 per credit) and the fewest credit hours (mean 29.0). Capstones and tracks were uncommon, and 10 of 46 (21.7%) of programs were accredited.
3. Cluster 3 (3/107, 2.8%): This cluster comprised in-person programs with an average of 37.3 credit hours and the highest tuition (mean US $748 per credit). These programs typically required a thesis and included AI-related tracks; accreditation was present in 1 of 3 (33.3%) cases.
4. Cluster 4 (54/107, 50.5%): The largest group, predominantly online programs, featured moderate tuition (mean US $514 per credit) and average credit loads (mean 35.0). Capstones were the dominant culminating experience, while tracks were rare, and this cluster contained the largest number of accredited programs (21/54, 38.9%).

## Discussion

### Principal Results

This cross-sectional analysis identified substantial variation in cost, structure, and curriculum across US graduate health informatics programs. Notably, accreditation was not associated with statistically significant differences in the tuition-normalized curriculum breadth structural proxy, credit requirements, or tuition per credit. We emphasize that tuition-normalized curriculum breadth is a structural proxy and should not be interpreted as a comprehensive assessment of program “value” or “quality,” as it does not incorporate instructional depth, total program cost, or postgraduation labor market outcomes.

Delivery format and internship requirements were more strongly associated with financial differences. Delivery format showed modest variation in credit requirements (95% CI 2.1‐17.3). This suggests that structural modality, rather than accreditation signaling, appears more closely associated with variation in the required educational investment.

Specifically, online nonaccredited programs required the fewest credits (17/107, 15.9%; mean 24.15, SD 9.89), while accredited online programs exhibited the highest tuition per credit. Furthermore, programs requiring internships were associated with significantly higher credit loads (mean 39.0, SD 8.1) than those without (mean 31.3, SD 10.7; *P*=.005). The lower credit requirements observed in the online nonaccredited sector may reflect structural program design differences such as accelerated residency requirements or stackable certificate-to-master’s pathways, where a 24-credit requirement represents the residency core completed after the transfer of prior graduate credits. Exploratory cluster analysis identified 4 program configurations, although we note that some configurations were rare (n≤4), suggesting that these configurations serve as descriptive representations of the current landscape rather than generalizable program models.

### Comparison With Prior Work

This study applies HCT to evaluate cost efficiency and curricular design in graduate health informatics. By analyzing tuition, accreditation, and curriculum across 107 programs, it assesses how institutions structure educational investments and whether these investments yield value in technical skill acquisition and affordability. This study provides a descriptive cross-sectional evaluation of US graduate health informatics programs using a multidimensional framework that integrates Value, Structure, and Curriculum.

The findings extend discussions on affordability, accreditation, and content alignment in health professions education and highlight persistent variation in how institutions operationalize value and quality. Tuition per credit varied widely, from below US $500 to above US $2000, with online nonaccredited programs showing the lowest credit requirements and accredited online programs the highest tuition per credit. This is consistent with reports that online programs can incur hidden or differential fees [5].

Accreditation status was not associated with differences in the tuition-normalized curriculum breadth structural proxy, aligning with literature suggesting that accreditation may support quality assurance without necessarily differentiating program cost efficiency. Accelerated pathways, such as combined bachelor’s-masters programs, were associated with lower tuition intensity**,** consistent with the premise that shorter time to completion can reduce the structural cost of skill acquisition [17].

### Implications for Education and Policy

#### Overview

Program structure varied widely across offerings, with capstone projects more common than theses, and internships were required in fewer than 20% of programs (21/107, 19.6%). Programs that required internships had higher average credit loads, suggesting increased curricular burden without corresponding tuition reduction. This pattern echoes prior research showing that unpaid placements can impose financial strain through lost income and indirect costs [18,19]; however, it is important to note that our data did not include student demographics, the paid or unpaid status of these placements, or the actual financial burden on individuals. Consequently, we offer the conjectural implication that internship experiences, while potentially valuable for applied learning, may inadvertently reinforce inequities among students with fewer financial resources or caregiving responsibilities.

The absence of a relationship between CAHIIM accreditation and either tuition-normalized curriculum breadth (*P*=.35; RQ2) or required credit hours (*P*=.39; RQ1) provides context regarding how accreditation aligns with structural program design. Although accreditation is frequently interpreted as an indicator of streamlined program design or stronger financial returns for students, these findings indicate that CAHIIM accreditation may function primarily as a signal of curricular credibility and baseline quality expectations [20] rather than as a structural determinant of program cost or duration. From the student viewpoint, CAHIIM accreditation was not associated with lower tuition-normalized curriculum breadth or required credit hours in this sample, within the constraints of this program-level descriptive analysis, suggesting that accreditation status alone does not differentiate this structural pricing-to-breadth proxy. While our study lacks outcome-level data to measure actual economic returns, this observed gap highlights the importance of more explicit communication regarding the scope and limits of what CAHIIM accreditation represents, particularly as concerns about affordability, debt, and workforce alignment increasingly inform graduate education choices.

Contrary to expectation, higher tuition was not associated with greater coverage of technology-focused content. Instead, programs with higher technology density tended to require more credits (*r*=0.21, *P*=.03), suggesting the need for broader curricular space to integrate competencies such as AI, data science, and cybersecurity. This supports calls for more robust technical training in health informatics curricula [9,14] and aligns with updated international recommendations emphasizing core informatics, data, and computer science competencies [9]. These findings indicate that tuition levels alone are not reliable indicators of curricular sophistication or alignment with emerging workforce needs.

#### AI Integration and Program Configurations

Beyond cost and accreditation, these findings suggest that graduate health informatics programs may consider integrating applied AI tools into their curricula. Systems for automated imaging, diagnostic support, and patient simulation tools, such as AI-driven diagnostics platforms like Smile.AI or automated smile analysis and orthodontic planning software like Dynasmile (developed by Chen et al [21,22]) and patient engagement platforms, illustrate emerging applications of AI for efficiency, assessment standardization, and patient engagement in clinical contexts. Embedding similar tools into curricula via case-based exercises and simulated workflows may provide opportunities for students to develop familiarity with evaluating, implementing, and managing AI technologies, including ethical and governance considerations. As upskilling remains a critical barrier to AI adoption in health information sectors [23], this hands-on exposure to applied AI tools could complement accreditation frameworks by emphasizing practical implementation alongside domain knowledge, without implying direct effects on workforce outcomes.

The 4 program configurations identified through cluster analysis (RQ8) reveal how graduate health informatics programs differ in how they strategically balance cost, curricular depth, and accreditation signaling. We posit as a conjectural interpretation that these configurations may correspond to distinct workforce pathways, although we emphasize that no student-level or employment outcome data were available to validate these associations.

For example, cluster 1 (high credit, high cost hybrid programs with capstone and thesis options; n=4) and cluster 3 (high tuition, in-person programs with thesis and advanced technical tracks; n=3) appear oriented toward research-intensive and technically advanced roles. Cluster 2 (low-cost, low-credit flexible programs; n=46) emphasizes rapid workforce entry and applied practice, while cluster 4 (moderately priced, predominantly online programs with capstone requirements; n=54) targets implementation-focused careers and professional advancement.

From a workforce perspective, program choice may reflect different career trajectories, yet these linkages remain theoretical associations rather than empirically measured results. Students may be selecting among differing program configurations, with each configuration potentially reflecting distinct role orientations within the health informatics labor market. Understanding these structural patterns may help describe how program design relates to targeted skill emphasis, although further research is required to link these configurations to actual labor market outcomes.

For prospective students, these configurations offer points of comparison regarding program choice. For institutions, they highlight considerations for evaluating credit requirements, aligning costs with curricular scope, and supporting equity in applied training opportunities. The tuition-normalized curriculum breadth metric may also serve as a practical descriptive indicator for internal dashboards, program review, and accreditation self-studies, particularly in the context of new federal requirements for program-level transparency under the Financial Value Transparency and Gainful Employment regulations [2].

### Future Research and Recommendations

The recommendations are as follows:

1. *Link program features to outcomes*: Pair program structure, tuition, and curriculum with graduate employment, earnings, and satisfaction data to test return on investment using sources such as College Scorecard and alumni surveys.
2. *Quantify hidden and modality-specific fees*: Prospectively capture mandatory online and program fees to refine tuition normalization and assess the true cost of online, hybrid, flexible, and in person formats.
3. *Evaluate equity impacts*: Examine whether internship requirements (21/107, 19.6%), accelerated pathways, and tuition levels differentially affect students by income, race and ethnicity, caregiving status, visa status, and employment status.
4. *Validate the tuition-normalized breadth metric*: Compare this metric with alternative measures that incorporate required credits, total program cost, and graduate outcomes. Test reliability across institutions and years, compare required-only with required plus elective domain coverage, and correlate the metric with external indicators of quality and outcomes.
5. *Deepen curriculum analytics*: Move beyond keyword counts to syllabus-level and assignment-level analyses that capture depth of AI, data science, and cybersecurity training and alignment with AMIA and International Medical Informatics Association competencies.
6. *Incorporate longitudinal designs*: Track cohorts over time to evaluate how changes in accreditation status, delivery format, or credit requirements influence affordability and outcomes.
7. *Conduct mixed methods studies*: Use interviews and focus groups with students, faculty, and program directors to contextualize quantitative patterns and identify barriers to transparency and affordability.
8. *Compare internationally*: Replicate the framework across countries to determine whether observed configurations are specific to the US tuition and regulatory environment or generalize to other contexts.
9. *Develop decision tools*: Translate findings into dashboards or checklists that help applicants compare programs on cost, structure, and curriculum, and that support institutional benchmarking and self-study.

### Limitations

This analysis relied on publicly reported data, which may omit hidden fees, special tuition categories, or real-world variation in student costs. The study focused on structural and curricular characteristics rather than outcomes such as employment, earnings, or satisfaction. Future work should triangulate program-level data with alumni surveys, employer input, and federal datasets to assess real-world outcomes, and consider cross-national comparisons to test generalizability.

Furthermore, we acknowledge that applying complex modeling (eg, ANCOVA and *k*-means clustering) to a relatively small, heterogeneous dataset of 107 programs carries risks of overfitting and spurious interpretation. Specifically, our clustering approach is based on mixed-data encodings analyzed using Euclidean distance, which may be less suitable for nominal variables. Although robustness checks using Ward’s hierarchical clustering, multiple internal validity indices, and bootstrap resampling supported the overall structure, the results should be interpreted as descriptive rather than definitive. The inclusion of very small clusters (n≤4) indicates that these specific program configurations are rare within the current landscape; they are presented for descriptive completeness but lack the statistical power to be generalizable or to support inferential claims.

### Conclusions

Graduate health informatics programs in the United States exhibit wide variation in cost, structure, and curriculum. Accreditation status was not associated with differences in the tuition-normalized curriculum breadth structural proxy in this program-level analysis, although accreditation was more prevalent within certain predominantly online program configurations. Accelerated formats and greater technical content emphasis (eg, AI and data science) were descriptively associated with lower credit requirements or broader curricular scope, suggesting potential pathways to align affordability with workforce-oriented training. Within an HCT lens, the structural cost of acquiring skills varies substantially and is not consistently aligned with common program design features. Program and policy strategies that more transparently align cost, accreditation signaling, and curricular emphasis may support efforts to promote equitable access to high-quality health informatics education.

## Supplementary material

10.2196/87479 Multimedia Appendix 1 Abbreviations.

10.2196/87479 Multimedia Appendix 2 Operational definitions.

10.2196/87479 Multimedia Appendix 3 Variable coding framework and data sources.

10.2196/87479 Multimedia Appendix 4 R (R Core Team) packages used in the study.

10.2196/87479 Multimedia Appendix 5 Descriptive characteristics of graduate health informatics programs (N=107).

10.2196/87479 Multimedia Appendix 6 ANCOVA results for tuition, program format, and accreditation predicting credit hour requirements.

10.2196/87479 Multimedia Appendix 7 Tukey post hoc pairwise contrasts for program format × accreditation.

10.2196/87479 Multimedia Appendix 8 Pairwise comparisons of tuition per credit by program format and accreditation status.

10.2196/87479 Multimedia Appendix 9 Tuition per credit by accelerated program status.

10.2196/87479 Multimedia Appendix 10 Tuition per credit by accelerated program status, stratified by Commission on Accreditation for Health Informatics and Information Management Education accreditation.

10.2196/87479 Multimedia Appendix 11 Pairwise comparisons of tuition per credit by program format.

10.2196/87479 Multimedia Appendix 12 Mean tuition per credit by program format and Commission on Accreditation for Health Informatics and Information Management Education accreditation.

10.2196/87479 Multimedia Appendix 13 Regression models testing technology content density and accreditation as predictors of tuition and credit load.

10.2196/87479 Multimedia Appendix 14 Crosstab of program format × timeline months.

10.2196/87479 Multimedia Appendix 15 Average credits by mandatory internship requirement.

10.2196/87479 Multimedia Appendix 16 Program duration distribution by program format.

10.2196/87479 Multimedia Appendix 17 Cluster profiles of graduate health informatics programs (N=107).

10.2196/87479 Multimedia Appendix 18 Commission on Accreditation for Health Informatics and Information Management Education accreditation by cluster.

10.2196/87479 Checklist 1 STROBE checklist for cross-sectional studies.

## Abbreviations

AI: artificial intelligence
AMIA: American Medical Informatics Association
CAHIIM: Commission on Accreditation for Health Informatics and Information Management Education
HCT: Human Capital Theory
IRB: institutional review board
STROBE: Strengthening the Reporting of Observational Studies in Epidemiology
