# Supplementary material for: Value, Structure, and Curriculum in US Graduate Health Informatics Programs: Cross-Sectional Study
Source: JMIR Med Educ. 2026 May 1;12:e87479. doi: 10.2196/87479 (PMC13134824; doi:10.2196/87479)
Supplement: Multimedia Appendix 1 [file mededu-v12-e87479-s001.docx]

**Multimedia Appendix 1.** Abbreviations.

| **Abbreviation** | **Full Term** |
| --- | --- |
| **AI** | Artificial Intelligence |
| **AMIA** | American Medical Informatics Association |
| **ANCOVA** | Analysis of Covariance |
| **BMI / BMHI** | Biomedical (and Health) Informatics |
| **CAHIIM** | Commission on Accreditation for Health Informatics and Information Management Education |
| **CS** | Computer Science |
| **F-1** | U.S. student visa category for academic studies |
| **HI** | Health Informatics |
| **IMIA** | International Medical Informatics Association |
| **IQR** | Interquartile Range |
| **IRB** | Institutional Review Board |
| **KPI** | Key Performance Indicator |
| **ML** | Machine Learning |
| **NA** | Not Available / Missing |
| **PSM** | Professional Science Master’s |
| **SD** | Standard Deviation |
| **STROBE** | Strengthening the Reporting of Observational Studies in Epidemiology |
