# Supplementary material for: Value, Structure, and Curriculum in US Graduate Health Informatics Programs: Cross-Sectional Study
Source: JMIR Med Educ. 2026 May 1;12:e87479. doi: 10.2196/87479 (PMC13134824; doi:10.2196/87479)
Supplement: Multimedia Appendix 2 [file mededu-v12-e87479-s002.docx]

**Multimedia Appendix 2.** Operational definitions.

| **Variable** | **Operational Definition / Coding Rule** |
| --- | --- |
| **Unit of analysis: Graduate program** | U.S.-based master’s or graduate-equivalent program in health/biomedical/clinical informatics or closely related healthcare analytics/data science marketed to the health sector, with publicly available curriculum and tuition information. |
| **Delivery format** | Online: fully online. In-person: primarily campus-based. Hybrid: planned mix. Flexible: program explicitly provides more than one delivery option for the same degree. |
| **Duration (months)** | Nominal time to completion as reported. Midpoint used when a range is listed. |
| **Total credits (hours)** | Required credits to complete degree. Midpoint used when a range is listed. |
| **Credit bands** | Very Low ≤20; Low 21–30; Typical 31–39; High 40–49; Very High ≥50. |
| **Tuition per credit (USD)** | Published tuition per credit hour. Semester/year tuition converted using published credit loads. Included: mandatory per-credit or per-term fees (e.g., online delivery fee). Excluded: optional fees (housing, insurance, parking). |
| **Total program cost (USD)** | Tuition per credit × total credits. Excludes optional fees unless mandated per credit. |
| **CAHIIM accreditation** | Yes/No — Status at time of collection; requires explicit, degree-specific confirmation on CAHIIM directory (excludes candidacy/department-only status).. |
| **F-1 visa eligibility** | Yes / Conditional / No. “Conditional” = limited intake or campus-only option required. |
| **Accelerated pathway** | Yes/No. Any formally advertised accelerated route (e.g., 3+2, 4+1, or other accelerated master’s). Subtypes recorded when specified. |
| **Professional Science Master’s (PSM)** | Yes/No. Program self-identifies as PSM or listed by PSM network. |
| **Culminating experience** | Capstone (required/optional); Thesis (required/optional); Internship (required/optional). “Both” = capstone and thesis both available. |
| **Tracks** | Yes/No. Named concentrations recorded (e.g., Data & Analytics; Clinical/Translational; Public Health/Leadership; Biomedical/Bioinformatics; Professional/Admin). |
| **Prerequisites** | Yes/No. Categories: Math/Stats; CS/Programming; Biological/Medical Sciences; Medical Terminology/HI; Other. |
| **Technology content density** | Count of predefined technology-related keywords. Measure of curricular emphasis and intent; does not measure syllabus depth or student skill acquisition. |
| **CAHIIM domain coverage (count)** | Number of distinct CAHIIM-aligned domains mapped to curriculum (leadership, data management, information systems, privacy/security, project management, standards/interoperability, analytics/decision support). |
| **Tuition-normalized curriculum breadth** | A structural ratio representing tuition per credit divided by the number of CAHIIM-aligned domains (USD per credit per domain). This is a descriptive measure of pricing intensity relative to curricular scope, not a measure of educational quality or student outcomes. |
| **Screening dispositions** | Duplicates (same program across sources), Ineligible (non-U.S., undergraduate/certificate-only, closed/suspended), or Incomplete (insufficient public info). |
| **Conflicting sources rule** | If catalog/brochure/website disagree, select the most recent dated source. If undated/tied, apply pre-specified coding rules or contact program. Archive URLs and access dates recorded. |
| **Missing data** | Encoded as NA. Analyses conducted with complete-case data per model; denominators reported. |
