# Supplementary material for: Value, Structure, and Curriculum in US Graduate Health Informatics Programs: Cross-Sectional Study
Source: JMIR Med Educ. 2026 May 1;12:e87479. doi: 10.2196/87479 (PMC13134824; doi:10.2196/87479)
Supplement: Multimedia Appendix 3 [file mededu-v12-e87479-s003.docx]

**Multimedia Appendix 3.** Variable coding framework and data sources.

| **Variable** | **Description / Recorded Value** | **Coding Scheme** | **Source of Information** | **Notes / Examples** |
| --- | --- | --- | --- | --- |
| Unit of analysis | Graduate program | One row per degree program | 3-Point Decision Matrix | Inclusion: (1) Health college affiliation, (2) Explicit "Health" title, OR (3) ≥3 health-specific core courses |
| Delivery format | Primary modality | Online / In-person / Hybrid / Flexible | Program website/catalog | **Flexible:** Coded when the same degree is explicitly marketed in ≥2 formats (e.g., Online and In-person tracks) |
| Duration (months) | Nominal time to completion | Integer; midpoint if range | Most recent dated source | E.g., “12–18 months” → 15 |
| Total credits | Required credits to graduate | Integer; midpoint if range | Catalog/plan of study | If tracks vary, use common/core requirement; note exceptions |
| Credit bands | Display grouping | ≤20; 21–30; 31–39; 40–49; ≥50 | Derived | Matches Tables in Results |
| Tuition per credit (USD) | Price per credit | Numeric USD | Convert term/year to per-credit | Includes mandatory per-credit fees; excludes optional fees (e.g., housing) |
| Total program cost (USD) | Tuition × credits | Numeric USD | Derived | Costs exclude optional fees unless mandated per credit |
| CAHIIM accreditation | Degree-specific status | Yes / No | CAHIIM/AMIA Directory | Verified at the specific degree level; excludes candidacy or department-only status. |
| F-1 visa eligibility | International support | Yes / Conditional / No | Program/international office pages | “Conditional” if campus-only or limited |
| Accelerated pathway | Reduced time/credits model | Yes / No (+ 3+2 / 4+1 / Other) | Program page | Formally advertised accelerated routes for credit reduction |
| PSM designation | Professional Science Master’s | Yes / No | Program/PSM network | Self-identified or listed |
| Culminating experience | Final degree requirement | Capstone / Thesis / Both / None; Internship (Y/N) | Curriculum pages | “Both” when capstone or thesis options exist |
| Internship Requirement | Applied learning | Yes / No | Curriculum pages | Specifically denotes a mandatory external placement |
| Tracks | Named concentrations | Yes / No + labels | Program pages | Data & Analytics; Clinical; Leadership; Bio/BMI; Admin |
| Prerequisites | Pre-enrollment requirements | Yes / No + categories | Admissions pages | Math/Stats; CS/Prog; Bio/Med; HI/MedTerm; Other |
| Tech content density | Technical emphasis score | Integer count of keywords | Keyword dictionary | Counts unique keywords across core/electives. Proxy for emphasis, not skill acquisition. |
| CAHIIM domain coverage | Breadth of domains | Integer (# domains) | Mapping rules | Domains listed in codebook |
| Screening disposition | Flow diagram status | Identified / Duplicates / Screened / Excluded / Included | Study search log | Provides the audit trail for Figure 2. |
| Conflicts resolution | Source precedence | Most recent dated source | Hierarchy Rule | If undated: priority given to Official Catalog over Marketing Brochure. |
| Missing data | Unavailable fields | NA | Rule | Complete-case per model; denominators reported |
| Tuition-normalized curriculum breadth | Structural cost ratio | (USD/credit) ÷ # domains | Derived from Value & Curriculum | A descriptive ratio of pricing relative to domain coverage |

**Multimedia Appendix 4:** Search Audit Trail

| **Component** | **Detailed Documentation for Replication** |
| --- | --- |
| Search Engine | Google Search (Private/Incognito mode to avoid algorithmic bias). |
| Exact Search Strings | (1) "graduate health informatics programs US"    (2) "health analytics masters US"    (3) "biomedical informatics master's degrees United States"    (4) "clinical informatics graduate curriculum US" |
| Search Depth | First 100 results per search string were screened. |
| Directories Seeded | CAHIIM Accredited Program Directory; AMIA Institutional Member Directory. |
| Data Collection Window | Finalized May 15, 2025. |

**Multimedia Appendix 4:** The 3-Point Decision Matrix (Inclusion Logic)

| **Criteria** | **Operational Rule for Inclusion** |
| --- | --- |
| 1. Institutional Affiliation | Degree is housed within a College of Medicine, Nursing, Public Health, or a dedicated Health Sciences department. |
| 2. Explicit Designation | The program title or official catalog explicitly lists a "Health," "Clinical," or "Medical" track/concentration. |
| 3. Curricular Core | Publicly available catalog requires $\geq$3 health-specific informatics courses (e.g., Electronic Health Records, Health Data Standards, Clinical Decision Support). |
