# Supplementary material for: Value, Structure, and Curriculum in US Graduate Health Informatics Programs: Cross-Sectional Study
Source: JMIR Med Educ. 2026 May 1;12:e87479. doi: 10.2196/87479 (PMC13134824; doi:10.2196/87479)
Supplement: Multimedia Appendix 4 [file mededu-v12-e87479-s004.docx]

**Multimedia Appendix 4.** R packages used in the study (R 4.4.1, May 2025).

| **Package** | **Version** | **Purpose / Role in Analysis** |
| --- | --- | --- |
| tidyverse | 2.0.0 | Core data science meta-package (dplyr, ggplot2, tidyr, readr, purrr, tibble, stringr, forcats) |
| knitr | 1.49 | Dynamic report generation and code chunk execution for R Markdown |
| apaTables | 2.0.8 | APA-style tables for statistical results |
| psych | 2.5.3 | Descriptives, reliability, and psychometrics utilities |
| janitor | 2.2.1 | Data cleaning helpers (e.g., clean_names, tabulations) |
| data.table | 1.17.6 | High-performance data manipulation |
| MASS | 7.3-65 | Classical statistics & datasets (e.g., modeling utilities) |
| kableExtra | 1.4.0 | Publication-ready tables (lightable styling in HTML/PDF/Word) |
| magrittr | 2.0.3 | Pipe operators and functional helpers |
| dplyr | 1.1.4 | Data manipulation verbs (select, filter, mutate, summarize) |
| purrr | 1.0.2 | Functional programming & list-column workflows |
| skimr | 2.2.1 | Quick, pretty dataset summaries |
| summarytools | 1.1.4 | Exploratory data summaries and crosstabs |
| emmeans | 1.11.2 | Estimated marginal means & contrasts (post-hoc) |
| WRS2 | 1.1-7 | Robust statistics (trimmed means, robust ANOVA/ANCOVA, etc.) |
| car | 3.1-3 | Regression/ANCOVA utilities (e.g., type-III SS), diagnostics |
| caret | 6.0-94 | Modeling workflow utilities (preprocess, resampling, tuning) |
| stringr | 1.5.1 | Consistent string processing |
| tidyr | 1.3.1 | Reshaping and tidy data tools |
| ggplot2 | 3.5.2 | Grammar-of-graphics plotting |
| factoextra | 1.0.7 | Visualization for multivariate analyses (PCA/clustering) |
| reshape2 | 1.4.4 | Legacy reshaping (melt/cast) |
| openxlsx | 4.2.8 | Read/write Excel .xlsx with formatting |
| writexl | 1.5.4 | Fast writing of Excel .xlsx |
