# Supplementary material for: Value, Structure, and Curriculum in US Graduate Health Informatics Programs: Cross-Sectional Study
Source: JMIR Med Educ. 2026 May 1;12:e87479. doi: 10.2196/87479 (PMC13134824; doi:10.2196/87479)
Supplement: Multimedia Appendix 5 [file mededu-v12-e87479-s005.docx]

**Multimedia Appendix 5.** Descriptive characteristics of graduate health informatics programs (N = 107).

| **Program Format** | **N (%)** |
| --- | --- |
| In-person | 23 (21.5%) |
| Online | 37 (34.6%) |
| Hybrid / Mixed | 32 (29.9%) |
| All Options Available (Flexible) | 15 (14.0%) |
| **Master’s Program Type** | **N (%)** |
| Health Informatics | 33 (30.8%) |
| Biomedical Informatics | 18 (16.8%) |
| Clinical Informatics | 15 (14.0%) |
| Healthcare Analytics & Data Science | 20 (18.7%) |
| Health IT, Security & Administration | 10 (9.3%) |
| Interdisciplinary / Leadership | 7 (6.5%) |
| Other / Mixed | 4 (3.7%) |
| **Program Timeline** | **N (%)** |
| ≤12 months | 19 (17.8%) |
| 13–18 months | 14 (13.1%) |
| 19–24 months | 72 (67.3%) |
| >24 months | 2 (1.9%) |
| **Credit Hours** | **N (%)** |
| Low (21–30) | 31 (29%) |
| Typical (31–39) | 53 (50%) |
| High (40–49) | 19 (18%) |
| Very High (50+) | 4 (4%) |
| **Tracks** | **N (%)** |
| No Tracks | 60 (56.1%) |
| With Tracks | 47 (43.9%) |
| – Data & Analytics | 15 (14.0%) |
| – Clinical / Translational | 12 (11.2%) |
| – Public Health / Leadership | 10 (9.3%) |
| – Biomedical / Bioinformatics | 6 (5.6%) |
| – Professional / Admin | 4 (3.7%) |
| **Prerequisites** | **N (%)** |
| Not Required | 72 (67.3%) |
| Required | 35 (32.7%) |
| – Math & Statistics | 20 (18.7%) |
| – Computer Science / Programming | 18 (16.8%) |
| – Biological / Medical Sciences | 12 (11.2%) |
| – Medical Terminology & Health Info | 7 (6.5%) |
| – Other Specialized | 4 (3.7%) |
| **Capstone / Thesis** | **N (%)** |
| Capstone only | 54 (50.5%) |
| Thesis only | 7 (7%) |
| Both options | 4 (4%) |
| Optional | 23 (21%) |
| None | 19 (17.8%) |
| **Internship Requirement** | **N (%)** |
| Yes | 21 (19.6%) |
| No | 86 (80.4%) |
| **Accelerated Pathways** | **N (%)** |
| Yes (Any) | 32 (29.9%) |
| – 3+2 programs | 7 (6.5%) |
| – 4+1 programs | 8 (7.5%) |
| – Other accelerated | 17 (15.9%) |
| No | 75 (70.1%) |
| **Tuition per Credit** | **N (%)** |
| Low (<$500) | 18 (16.8%) |
| Moderate ($500–999) | 42 (39.3%) |
| High ($1,000–1,999) | 29 (27.1%) |
| Premium ($2,000+) | 14 (13.1%) |
| Unclear / Mixed | 4 (3.7%) |
| **PSM Designation** | **N (%)** |
| Yes | 7 (6.5%) |
| No | 100 (93.5%) |
| **F1 Visa Support** | **N (%)** |
| Yes | 63 (58.9%) |
| Conditional Yes | 3 (2.8%) |
| No | 41 (38.3%) |
| **CAHIIM Accreditation** | **N (%)** |
| Yes | 33 (31%) |
| No | 47 (69%) |
