# Supplementary material for: Value, Structure, and Curriculum in US Graduate Health Informatics Programs: Cross-Sectional Study
Source: JMIR Med Educ. 2026 May 1;12:e87479. doi: 10.2196/87479 (PMC13134824; doi:10.2196/87479)
Supplement: Multimedia Appendix 6 [file mededu-v12-e87479-s006.docx]

**Multimedia Appendix 6.** ANCOVA results for tuition, program format, and accreditation predicting credit hour requirements.

| **Predictor** | **Sum of Squares** | **df** | **F** | **p-value** |
| --- | --- | --- | --- | --- |
| Tuition (covariate) | 209.9 | 1 | 1.93 | 0.168 |
| Program Format | 883.4 | 3 | 2.70 | 0.049 * |
| CAHIIM Accreditation | 0.7 | 1 | 0.01 | 0.935 |
| Program Format × Accreditation | 305.6 | 3 | 0.93 | 0.427 |
| Residuals | 10680.4 | 98 |  |  |

p *< .05 indicated with an asterisk (*).*
