# Supplementary material for: Value, Structure, and Curriculum in US Graduate Health Informatics Programs: Cross-Sectional Study
Source: JMIR Med Educ. 2026 May 1;12:e87479. doi: 10.2196/87479 (PMC13134824; doi:10.2196/87479)
Supplement: Multimedia Appendix 7 [file mededu-v12-e87479-s007.docx]

### **Multimedia Appendix 7. Tukey post-hoc pairwise contrasts for program format × accreditation.**

| **Contrast** | **Estimate** | **SE** | **df** | **t-ratio** | **P value** |
| --- | --- | --- | --- | --- | --- |
| Flexible No: Hybrid No | 0.53 | 5.82 | 98 | 0.09 | >.99 |
| Flexible No: In-person No | -0.04 | 5.29 | 98 | -0.01 | >.99 |
| Flexible No: Online No | 9.93 | 5.49 | 98 | 1.81 | 0.62 |
| Flexible No: Flexible Yes | 0.42 | 5.17 | 98 | 0.08 | >.99 |
| Hybrid No: In-person No | -0.56 | 4.26 | 98 | -0.13 | >.99 |
| Hybrid No: Online No | 9.4 | 4.53 | 98 | 2.08 | 0.44 |
| In-person No: Online No | 9.97 | 3.82 | 98 | 2.61 | 0.17 |
| Online No: Flexible Yes | -9.51 | 3.65 | 98 | -2.6 | 0.17 |
| Online No: Hybrid Yes | -12.13 | 4.89 | 98 | -2.48 | 0.22 |
| Online No: Online Yes | -8.19 | 3.61 | 98 | -2.27 | 0.32 |
| Flexible Yes: Online Yes | 1.32 | 3.09 | 98 | 0.43 | >.99 |

Note: p-values adjusted with Tukey’s method for multiple comparisons.
