# Supplementary material for: Value, Structure, and Curriculum in US Graduate Health Informatics Programs: Cross-Sectional Study
Source: JMIR Med Educ. 2026 May 1;12:e87479. doi: 10.2196/87479 (PMC13134824; doi:10.2196/87479)
Supplement: Multimedia Appendix 8 [file mededu-v12-e87479-s008.docx]

**Multimedia Appendix 8.** Pairwise comparisons of tuition per credit by program format and accreditation status.

| **Comparison (Format × Accreditation)** | **Mean Diff** | **95% CI (Lower, Upper)** | **P adj** |
| --- | --- | --- | --- |
| Hybrid:No – Flexible:No | 65.76 | -704.13, 835.64 | >.99 |
| In-person:No – Flexible:No | 131.77 | -566.00, 829.53 | >.99 |
| Online:No – Flexible:No | -35.47 | -761.82, 690.88 | >.99 |
| Flexible:Yes – Flexible:No | -29.38 | -713.21, 654.46 | >.99 |
| Hybrid:Yes – Flexible:No | -11.85 | -820.05, 796.36 | >.99 |
| In-person:Yes – Flexible:No | -133.61 | -969.41, 702.19 | >.99 |
| Online:Yes – Flexible:No | 294.79 | -377.22, 966.80 | .87 |
| Online:Yes – In-person:Yes | 428.40 | -356.12, 1212.92 | .68 |

Note: *Table truncated; full results available upon request or in complete dataset.*
