# Supplementary figures and images for: Value, Structure, and Curriculum in US Graduate Health Informatics Programs: Cross-Sectional Study
Source: JMIR Med Educ. 2026 May 1;12:e87479. doi: 10.2196/87479 (PMC13134824; doi:10.2196/87479)

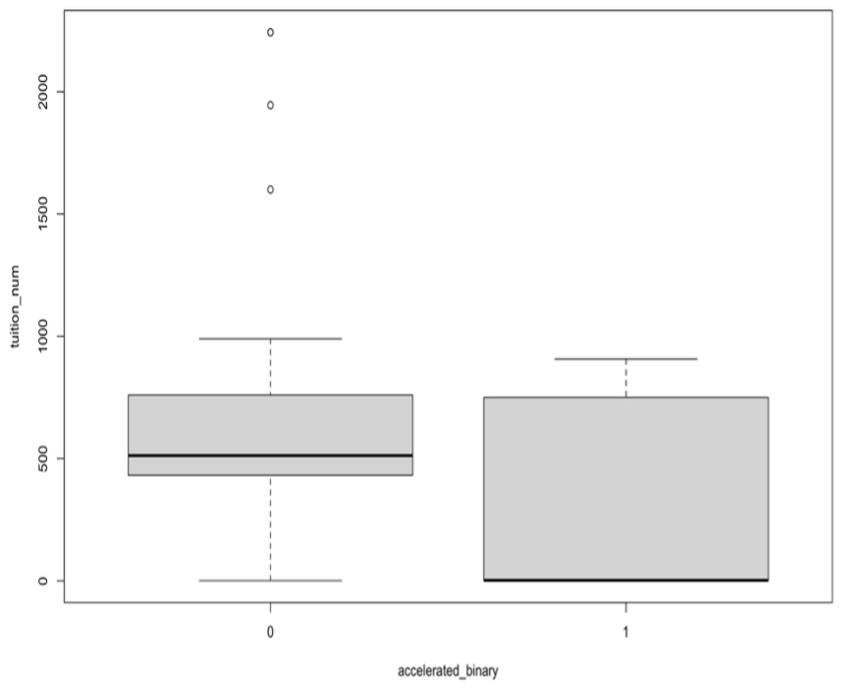

Supplement: Multimedia Appendix 9 [file mededu-v12-e87479-s009.png]

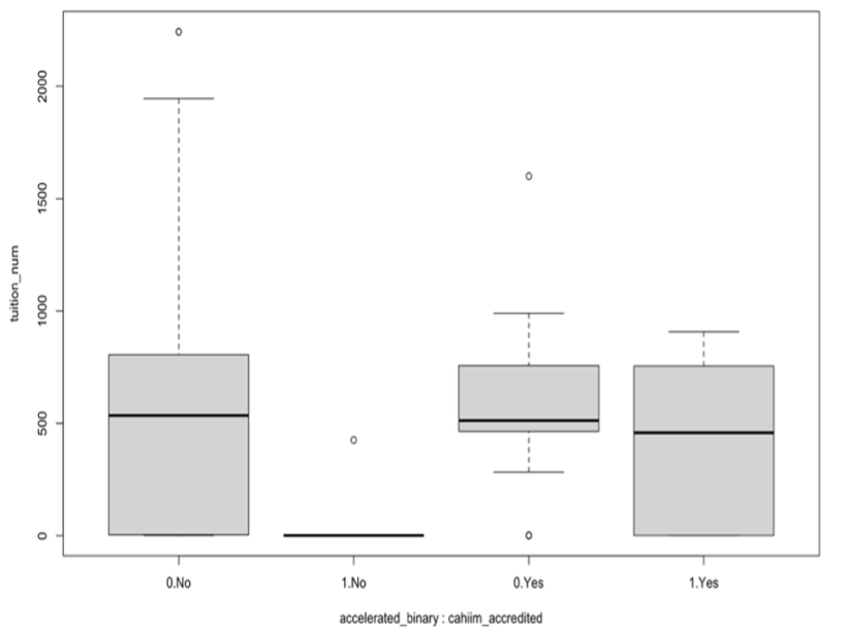

Supplement: Multimedia Appendix 10 [file mededu-v12-e87479-s010.png]

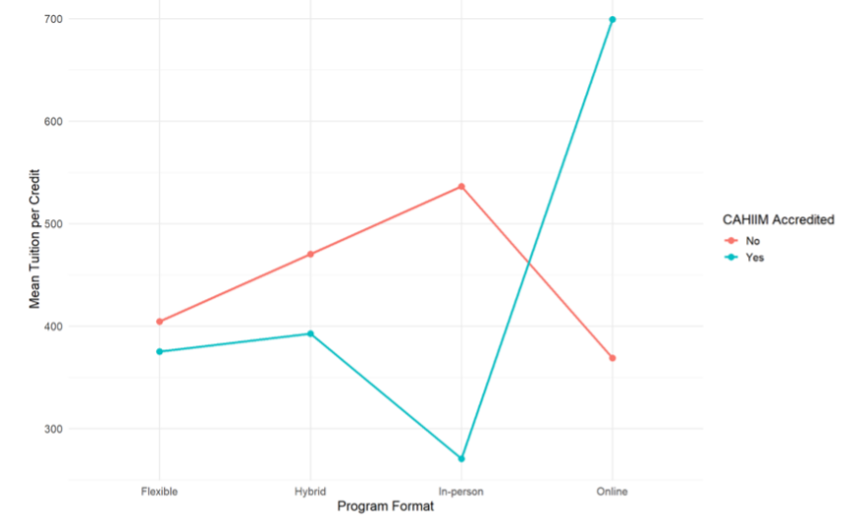

Supplement: Multimedia Appendix 12 [file mededu-v12-e87479-s012.png]

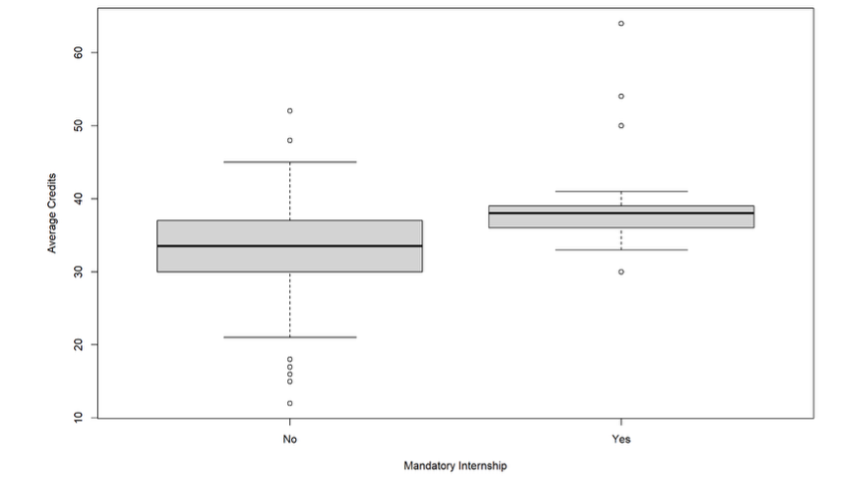

Supplement: Multimedia Appendix 15 [file mededu-v12-e87479-s015.png]

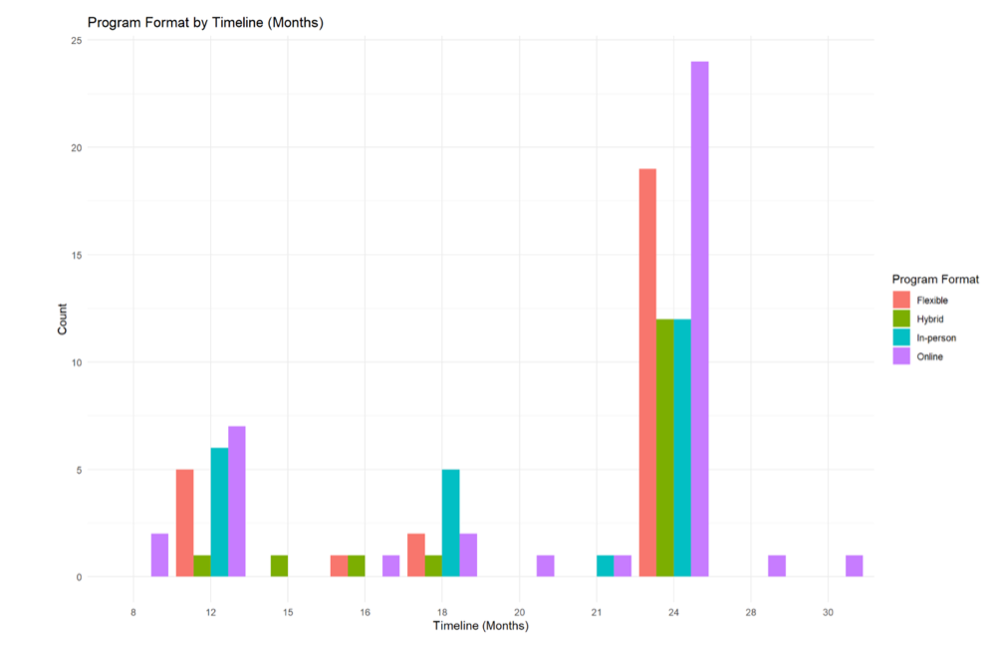

Supplement: Multimedia Appendix 16 [file mededu-v12-e87479-s016.png]
