# Supplementary material for: Value, Structure, and Curriculum in US Graduate Health Informatics Programs: Cross-Sectional Study
Source: JMIR Med Educ. 2026 May 1;12:e87479. doi: 10.2196/87479 (PMC13134824; doi:10.2196/87479)
Supplement: Multimedia Appendix 11 [file mededu-v12-e87479-s011.docx]

**Multimedia Appendix 11.** Pairwise comparisons of tuition per credit by program format.

| **Comparison (Program Format)** | **Mean Diff** | **95% CI (Lower, Upper)** | **P adj** |
| --- | --- | --- | --- |
| Hybrid – Flexible | 55.74 | -311.73, 423.22 | .98 |
| In-person – Flexible | 89.36 | -237.41, 416.12 | .89 |
| Online – Flexible | 211.39 | -78.72, 501.50 | .23 |
| In-person – Hybrid | 33.62 | -342.31, 409.54 | .99 |
| Online – Hybrid | 155.65 | -188.89, 500.19 | .64 |
| Online – In-person | 122.03 | -178.71, 422.77 | .71 |

***Note****: Differences are based on Tukey-adjusted post-hoc tests.
CI = Confidence Interval;* p *adj = Adjusted* p*-value for multiple comparisons.*
