# Supplementary material for: Value, Structure, and Curriculum in US Graduate Health Informatics Programs: Cross-Sectional Study
Source: JMIR Med Educ. 2026 May 1;12:e87479. doi: 10.2196/87479 (PMC13134824; doi:10.2196/87479)
Supplement: Multimedia Appendix 13 [file mededu-v12-e87479-s013.docx]

**Multimedia Appendix 13.** Regression models testing technology content density and accreditation as predictors of tuition and credit load.

| **Predictor** | **β (Estimate)** | **SE** | **t** | **p** |
| --- | --- | --- | --- | --- |
| **Model 1. Tuition per Credit** |  |  |  |  |
| Intercept | 816.85 | 121.59 | 6.72 | <.001 |
| Tech density | –10.25 | 17.58 | –0.58 | .561 |
| Accreditation (Yes = 1) | –120.59 | 153.41 | –0.79 | .434 |
| Tech density × Accreditation | –1.24 | 22.69 | –0.06 | .957 |
| Model fit: R² = .029, adj. R² = .001, F(3, 103) = 1.02, p = .385 |  |  |  |  |
| **Model 2. Credit Load (Hours)** |  |  |  |  |
| Intercept | 24.56 | 2.94 | 8.35 | <.001 |
| Tech density | 1.16 | 0.43 | 2.74 | .007 |
| Accreditation (Yes = 1) | 7.77 | 3.71 | 2.09 | .039 |
| Tech density × Accreditation | –0.88 | 0.55 | –1.60 | .113 |
| Model fit: R² = .085, adj. R² = .059, F(3, 103) = 3.21, p = .026 |  |  |  |  |
