# Supplementary material for: Value, Structure, and Curriculum in US Graduate Health Informatics Programs: Cross-Sectional Study
Source: JMIR Med Educ. 2026 May 1;12:e87479. doi: 10.2196/87479 (PMC13134824; doi:10.2196/87479)
Supplement: Multimedia Appendix 14 [file mededu-v12-e87479-s014.docx]

**Multimedia Appendix 14.** Crosstab of program format × timeline months.

| **Timeline (Months)** | **Flexible (n=15)** | **Hybrid (n=32)** | **In-person (n=23)** | **Online (n=37)** | **Total (N=107)** |
| --- | --- | --- | --- | --- | --- |
| 8 | 0 | 0 | 0 | 2 | 2 |
| 12 | 2 | 1 | 4 | 7 | 14 |
| 15–16 | 1 | 2 | 0 | 1 | 4 |
| 18 | 2 | 1 | 5 | 2 | 10 |
| 20–21 | 0 | 0 | 1 | 2 | 3 |
| 24 | 10 | 28 | 13 | 21 | 72 |
| >24 | 0 | 0 | 0 | 2 | 2 |
| Total | 15 | 32 | 23 | 37 | 107 |
