# Supplementary material for: Value, Structure, and Curriculum in US Graduate Health Informatics Programs: Cross-Sectional Study
Source: JMIR Med Educ. 2026 May 1;12:e87479. doi: 10.2196/87479 (PMC13134824; doi:10.2196/87479)
Supplement: Multimedia Appendix 17 [file mededu-v12-e87479-s017.docx]

**Multimedia Appendix 17.** Cluster profiles of graduate health informatics programs (N = 107).

| **Cluster** | **n** | **Avg. Credits (Mean)** | **Avg. Tuition (USD/credit)** | **Dominant Modality** | **Primary Culminating Experience** | **Common Tracks** | **% Accredited** |
| --- | --- | --- | --- | --- | --- | --- | --- |
| **1** | 4 | 43 | $668 | Hybrid | Both (Capstone & Thesis) | Research-intensive | **25%** |
| **2** | 46 | 29 | $424 | Flexible | None / Optional | Workforce-applied | **22%** |
| **3** | 3 | 37 | $748 | In-person | Thesis | Advanced Technical (AI) | **33%** |
| **4** | 54 | 35 | $514 | Online | Capstone | Implementation-focused | **39%** |

Note. Clusters derived from k-means analysis using program format, credits, tuition, culminating experience, and track offerings
