# Supplementary material for: Value, Structure, and Curriculum in US Graduate Health Informatics Programs: Cross-Sectional Study
Source: JMIR Med Educ. 2026 May 1;12:e87479. doi: 10.2196/87479 (PMC13134824; doi:10.2196/87479)
Supplement: Multimedia Appendix 18 [file mededu-v12-e87479-s018.docx]

**Multimedia Appendix 18.** **CAHIIM accreditation by cluster.**

| **Cluster** | **CAHIIM Accreditation** | **n** | **% within Cluster** |
| --- | --- | --- | --- |
| 1 | Yes | 1 | 25% |
|  | No | 3 | 75% |
| 2 | Yes | 10 | 22% |
|  | No | 36 | 78% |
| 3 | Yes | 1 | 33% |
|  | No | 2 | 67% |
| 4 | Yes | 21 | 39% |
|  | No | 33 | 61% |
| Total | Yes | 33 | 31% |

Note. Percentages are calculated within cluster.
