# Supplementary material for: Value, Structure, and Curriculum in US Graduate Health Informatics Programs: Cross-Sectional Study
Source: JMIR Med Educ. 2026 May 1;12:e87479. doi: 10.2196/87479 (PMC13134824; doi:10.2196/87479)
Supplement: Checklist 1 [file mededu-v12-e87479-s019.docx]

# **Checklist 1.** STROBE (Strengthening the Reporting of OBservational studies in Epidemiology) checklist for cross-sectional studies.

| **Checklist Item** | **Item No.** | **Description** |
| --- | --- | --- |
| Title and abstract | 1 | Indicate the study’s design with a commonly used term and provide an informative summary. |
| Background/rationale | 2 | Explain the scientific background and rationale for the study. |
| Objectives | 3 | State specific objectives, including any prespecified hypotheses. |
| Study design | 4 | Present key elements of the study design early in the paper. |
| Setting | 5 | Describe the setting, locations, and relevant dates, including data collection periods. |
| Participants | 6 | Give eligibility criteria and the sources and methods of participant selection. |
| Variables | 7 | Clearly define outcomes, exposures, predictors, potential confounders, and effect modifiers. |
| Data sources/measurement | 8 | For each variable of interest, give data sources and details of assessment. |
| Bias | 9 | Describe efforts to address potential sources of bias. |
| Study size | 10 | Explain how the study size was arrived at. |
| Quantitative variables | 11 | Explain how quantitative variables were handled in analyses. |
| Statistical methods | 12 | Describe all statistical methods used, including those for controlling confounding. |
| Participants (Results) | 13 | Report numbers of individuals at each stage of study and reasons for non-participation. |
| Descriptive data | 14 | Provide characteristics of participants and information on exposures and potential confounders. |
| Outcome data | 15 | Report numbers of outcome events or summary measures. |
| Main results | 16 | Give unadjusted and adjusted estimates with precision. |
| Other analyses | 17 | Report other analyses done, such as subgroup or sensitivity analyses. |
| Key results | 18 | Summarize key results with reference to study objectives. |
| Limitations | 19 | Discuss limitations, including potential bias and imprecision. |
| Interpretation | 20 | Provide a cautious interpretation considering study limitations and other evidence. |
| Generalisability | 21 | Discuss the generalisability of the study results. |
| Funding | 22 | Give funding sources and the role of the funders. |
